# A method for the prediction of GPCRs coupling specificity to G-proteins using refined profile Hidden Markov Models

**DOI:** 10.1186/1471-2105-6-104

**Published:** 2005-04-22

**Authors:** Nikolaos G Sgourakis, Pantelis G Bagos, Panagiotis K Papasaikas, Stavros J Hamodrakas

**Affiliations:** 1 Department of Cell Biology and Biophysics, Faculty of Biology, University of Athens, Athens 157 01, Greece

## Abstract

**Background:**

G- Protein coupled receptors (GPCRs) comprise the largest group of eukaryotic cell surface receptors with great pharmacological interest. A broad range of native ligands interact and activate GPCRs, leading to signal transduction within cells. Most of these responses are mediated through the interaction of GPCRs with heterotrimeric GTP-binding proteins (G-proteins). Due to the information explosion in biological sequence databases, the development of software algorithms that could predict properties of GPCRs is important. Experimental data reported in the literature suggest that heterotrimeric G-proteins interact with parts of the activated receptor at the transmembrane helix-intracellular loop interface. Utilizing this information and membrane topology information, we have developed an intensive exploratory approach to generate a refined library of statistical models (Hidden Markov Models) that predict the coupling preference of GPCRs to heterotrimeric G-proteins. The method predicts the coupling preferences of GPCRs to G_s_, G_i/o _and G_q/11_, but not G_12/13 _subfamilies.

**Results:**

Using a dataset of 282 GPCR sequences of known coupling preference to G-proteins and adopting a five-fold cross-validation procedure, the method yielded an 89.7% correct classification rate. In a validation set comprised of all receptor sequences that are species homologues to GPCRs with known coupling preferences, excluding the sequences used to train the models, our method yields a correct classification rate of 91.0%. Furthermore, promiscuous coupling properties were correctly predicted for 6 of the 24 GPCRs that are known to interact with more than one subfamily of G-proteins.

**Conclusion:**

Our method demonstrates high correct classification rate. Unlike previously published methods performing the same task, it does not require any transmembrane topology prediction in a preceding step. A web-server for the prediction of GPCRs coupling specificity to G-proteins available for non-commercial users is located at .

## Background

G-protein coupled receptors are important receivers of information input to eukaryotic cells. They share a common fold of seven transmembrane helices arranged as a seven *α*-helix bundle, as confirmed by analysis of the crystal structure of Rhodopsin [1] that has been extensively used as template for homology-based modeling of GPCRs [2-4]. A collection of messages of extreme diversity including photons and native agonists, such as ions, odorants and pheromones, amino acids, nucleotides, peptides, biogenic amines, prostaglandines and glycoprotein hormones [5] interact with different extracellular and/or transmembrane domains of GPCRs, in order to convey their messages to the interior of the cell [2,6]. Based primarily on shared sequence motifs, six distinct families of GPCRs are traditionally defined: A, B, C, D, E and the frizzled/smoothened family, as summarized in the GPCRDB classification scheme [7]. Various methods have been deployed for higher-level classification of GPCRs including profile Hidden Markov Models [8,9], support vector machines [10] and Position Specific Scoring Matrices [11].

The physiological response of the interaction between a GPCR and one of its ligands is judged by the subset of the inactive heterotrimeric (*αβγ*) G-proteins within the cell that interact with the activated receptor complex, although many receptors mediate their actions through G-protein independent signaling pathways [2]. Different agonists may stabilize complexes of GPCRs with G-proteins belonging to different subfamilies (G_s_, G_i/o_, G_q/11 _or G_12/13_) resulting in the activation of different signaling pathways [12].

G-proteins are heterotrimeric complexes, named after their *α *subunits. On a basis of sequence identity, at least 16 discrete *α *subunits have been identified and classified into four subfamilies: G_s _and G_i/o_, which stimulate and inhibit respectively adenylate cyclase, G_q/11 _which stimulate phospholipase C, and the less characterized G_12/13 _subfamily that activate the Na+/H+ exchanger pathway [13-17]. We should mention at this point, that in the gpDB classification [18], the term "families" has been reserved for this level of hierarchy of G proteins, however hereinafter we will use the term "subfamilies" instead.

Agonist binding to GPCRs leads to association of the heterotrimeric G-protein with the receptor, which triggers the exchange of the guanosine diphosphate (GDP) bound on the *α*-subunit of the G-protein with guanosine triphosphate (GTP). These events promote the dissociation of the *α *subunit of the G-protein from the receptor and the *βγ *complex. The dissociated subunits can activate or inhibit several effector proteins, such as adenylyl cyclase 1–9, PLC*β *1–4, tyrosine kinases, ion channels and molecules of the mitogen-activated protein kinase pathway, resulting in a variety of cellular functions that depend on the biological specificity of the dissociated subunits [17,19]. G-protein *α *subunits possess an intrinsic GTPase activity, which enables them to act as time switches: Hydrolysis of the bound GTP to GDP promotes the re-association of the *α *subunit with the *βγ *dimer and renders the G-protein in an inactive form.

Due to the lack of structural data for activated GPCR complexes, several complementary approaches have been used to decipher the molecular events leading to G-protein activation, and to identify the regions that determine the coupling specificity of a GPCR to a subset of the pool of intracellular G-proteins. These biochemical approaches, that were focused mainly on A GPCRs, include site-directed mutagenesis studies [20], chimeric receptor engineering [21,22], the use of synthetic peptides to mimic the GPCR regions that activate G-proteins [23] and antibodies to neutralize GPCR binding sites on the G-proteins [24,25]. These studies revealed the major role of GPCR intracellular loops, especially the second and third, and the C-terminal region, as the main determinants of GPCR coupling specificity. Furthermore, structural data from high resolution X-ray diffraction of the light-sensing GPCR rhodopsin, as well as complementary methods (Nuclear Magnetic Resonance Spectroscopy, Electron Spin Resonance Spectroscopy, protein engineering, amino acid fluorescent replacement) [26-28] have indicated that ligand binding induces large conformational changes. These conformational changes reveal GPCR regions buried within the membrane which could interact with the G-protein [5]. Through a combination of entropy variability plots and correlated mutation analysis, key residues for a variety of GPCR functions, including coupling to G-proteins, can be identified and a mechanism of GPCR activation has been proposed [29-31].

Due to their role as information receivers of eukaryotic cells, GPCRs are involved in many pathophysiological responses. They comprise attractive drug targets for a variety of diseases, including cancer [32], Alzheimer's syndrome [33] and AIDS [34]. Indeed, over 50% of all prescribed drugs target on GPCRs [35]. Furthermore, the information explosion in biological sequence databases has resulted in many GPCR entries of unknown ligand binding properties, known as orphan receptors. In order to screen these orphan receptors with libraries of potential ligands, researchers must be able to assay the GPCR-ligand interaction through a downstream event. Such events are transcription of a reporter gene or rise in second messenger concentration, which is dependent on the interaction of the GPCR under study with members of a specific G-protein subfamily. Thus, knowing or being able to predict, the coupling specificity of orphan GPCRs to G-protein subfamilies, is essential for choosing the appropriate cell lines for heterologous expression and any further in vitro and in vivo studies of potential drug targets [36]. Meanwhile, a dataset of GPCRs of known coupling specificity exists [37], large enough to guide an in silico database mining approach that could aid further in vivo GPCR research. Furthermore, in a work published recently, many GPCRs and their interactions with G-proteins have been summarized in the gpDB system [18].

As in every biological interaction, the specificity of GPCR coupling to specific G-proteins is determined by structural components located on contact regions of the molecules. Since the three-dimensional architecture of a protein is encoded in protein sequence, GPCR coupling specificity could be defined by sequence alone. However, GPCRs with low sequence similarity may couple to members of the same subfamily of G-proteins, while members of the same GPCR subfamilies often couple to members of distinct G-protein subfamilies [38]. In addition, GPCR coupling is not a one-by-one function since many GPCRs, known as promiscuous GPCRs, have been proven to couple to members of more than one G-protein subfamilies. Due to these limitations, GPCR coupling specificity prediction in one step using sequence comparison methods such as the BLAST [39] or CLUSTALW [40] algorithms is insufficient [36]. However a weak sequence signal can be detected among receptor subfamilies where G- protein selectivity was a recent evolutionary process, such as the biogenic amines receptors [41].

Previous computational methods of GPCR coupling specificity to G-protein subfamilies have been applied on *a priori *selected intracellular regions of GPCR sequences. A Naive Bayes model [42] yields a 72% correct classification rate, while a data-mining approach that combined pattern discovery with membrane topology prediction [43] has also been applied in an effort to model GPCR regions that determine coupling specificity. However, previous approaches are either context-dependent on the *a priori *knowledge that GPCR coupling specificity is governed by the entire intracellular regions sequence or limited by the non-probabilistic nature and limited descriptive power of patterns as regular expressions, that cannot implement weights to different sequence variation. The approach of this study is exploratory regarding the length and localization of the coupling determining regions among the intracellular regions sequences and recruits profile Hidden Markov Models (pHMMs) as highly discriminative models of biological sequences that have a formal probabilistic basis [44]. The results obtained by this method, presented below, justify the chosen approach.

## Results and discussion

Our primary aim was to develop a wide-range predictive system that can be applied with the same discriminative power globally, for all three main GPCR coupling groups, being also able to model promiscuous receptor coupling. Our method proved to be self-consistent: Using a set of 282 GPCR sequences of experimentally identified coupling properties, according to the Trends in Pharmacological Sciences nomenclature supplement of receptors and ion channels (TiPS) [37], that were used to train the models and adopting a five-fold cross-validation procedure, the methods yielded a 89.7% correct classification rate. When tested in 479 sequences of GPCRs (retrieved also from the UniProt database [45]) that are homologous to the sequences used to train the models and whose coupling properties are also summarized in [37], at a subtype level, our method yields a 91.0% correct classification rate (Table 1). Finally, the method predicts correctly the coupling specificity of 25 out of 30 GPCRs derived from the gpDB database [18] that were not included in [37] (Table 2).

In order to assess the efficiency of the same method trained on a smaller and non-redundant dataset, the same procedure was applied to a dataset containing only the human GPCRs in the original training set. Alternative pHMMs were generated and integrated into a second predictive system that proved to be also self-consistent. On this human-only dataset, correct classification rate in a five-fold cross-validation, is 86% (data not shown). When these models were applied to the 479 sequences of the validation set, the correct classification rate was 88.9%, showing an insignificant decrease, as one would expect for a non-overfitted method. Additionally, when the model that was trained on human sequences, was applied to the remaining 178 non-human sequences derived from [37], yields also a high correct classification rate of 88.8%.

Due to insufficient experimental data, resulting in uncertainty about whether or not most receptors that are known to couple with a specific G-protein group can couple with G-proteins of another subfamily under different physiological conditions, we cannot estimate whether all of the promiscuous predictions are correct or not. For instance, a GPCR that is reported to couple only to G-proteins members of G_i/o _subfamily, may proved that couples also to members of G_s _subfamily. It is also well-known that the same GPCR may also couple to different G-protein subfamilies in different heterogenous expression systems. Promiscuous coupling was correctly predicted for 6 out of 24 GPCRs of known promiscuous coupling properties according to information in [37], as one can observe in Table 3. We did not attempt to train any pHMMs from sequences that have been proven to be promiscuous, in order to avoid unnecessary complexity and unequal distribution of the training set to the three major coupling groups of GPCRs.

The main reason that no pHMMs have been constructed that indicate coupling to G_12/13 _proteins is the limited amount of data available for the coupling properties of this subfamily of G-proteins. For this reason, this feature is not provided by any of the already published methods that perform the same task. Furthermore, to the knowledge of the authors no promiscuous GPCRs are included in the training set (i.e. GPCRs that couple to members from multiple subfamilies of G-proteins), and no receptors that preferentially couple only to members of the G_12/13 _subfamily have been identified [2]. Therefore, constructing pHMMs that classify G_12/13 _coupled GPCRs with high discriminative power, at this moment, is practically impossible. Once larger datasets have been established in the future, promiscuous receptors could be included in the training set, allowing predictions for G_12/13 _coupled receptors.

Our exploratory approach resulted in the discovery of sub-regions within the intracellular GPCR domains that play a key role in determining GPCR coupling specificity to G-proteins. The contribution of these regions to the overall coupling scheme of GPCRs could arise through short-range protein-protein interactions with their structural counterparts in G-proteins, that is, through intermolecular stabilizing interactions that enable several regions of the GPCR molecule to interact with G-proteins. The conformation of the intracellular regions of GPCRs is regulated by intramolecular interactions between the intracellular segments [38]. Furthermore, each query against the refined library of pHMMs reveals regions of high identity to the profiles, if such exist in the target sequence. Residues in these identified intracellular regions could be targeted for site-directed mutagenesis approaches in order to elucidate the structural features of GPCR – G-protein coupling.

Our method can only predict the potential of interaction between a GPCR and a G-protein subfamily, since its only input is the GPCR sequence. Thus, common in vivo regulators of GPCR coupling specificity including mechanisms such as selective targeting of GPCRs to specific cell-membrane regions, post-translational modifications [46,47] or effects of accessory/scaffolding proteins interacting with GPCRs (reviewed in [2]) cannot be modeled by our prediction system. Also, GPCR homo- or hetero-dimerization, that appears to be a common feature of many GPCRs, necessary for G-protein activation [48-50] cannot be directly included in our prediction system.

pHMMs derived from this study have been trained to model sub-regions within GPCR intracellular domains rather than entire GPCR sequences. The *a priori *knowledge that a query sequence belongs to a GPCR would be valuable in enhancing the predictive power of the method. When the method is applied to the non-GPCR receptor and the globular protein non-redundant test sets, it produces false positives with a rate 19.2% and 6.4% respectively. However filtering the query sequences, by using 7-transmembrane domain pHMMs derived from the Pfam database Version 14.0 [51] in a preceding step, diminishes completely the above false positive results without affecting the overall sensitivity of the method. All six pHMMs for 7-transmembrane receptors contained in the Pfam database Version 14.0 have been integrated into our publicly available method. In conclusion, the method could effectively be used in combination with existing 7-transmembrane receptor predictive systems for genome-wide applications.

**Figure 1:**
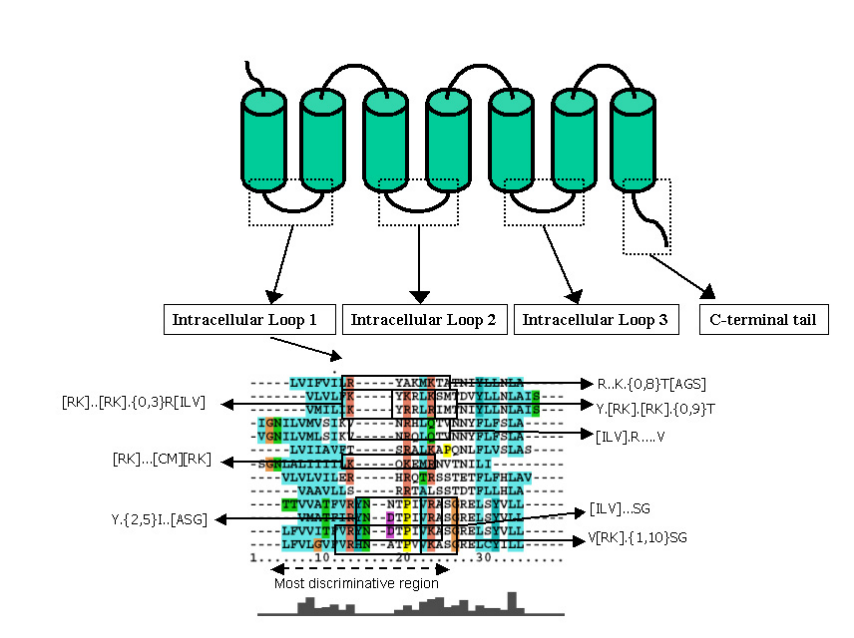
Comparison of the methods. Coupling determining patterns discovered by Moller et al. [43], show redundancy in their targets, since different patterns may apply to the same sequences in an overlapping manner. In addition, there are loop sequences of major GPCR subfamilies not characterized by any pattern, possibly due to low sequence identity. In comparison to patterns, profile Hidden Markov models (pHMMs) provide a whole sequence scoring scheme. Sequence information contained in multiple patterns can be integrated in a single pHMM derived from a low entropy region of a multiple sequence alignment. Thus, every query sequence can be given a score that has a formal probabilistic interpretation.

Compared to other previously published methods, performing the same task, our method does not only perform significantly better in terms of overall accuracy, but also employs additional superior features. Firstly, it does not rely on the identification of intracellular loops as does the Naive Bayes method in [42]. Our method was trained using the annotations for the transmembrane regions (which in most cases come from prediction methods) but in the testing phase no such information is required, thus it operates using as input solely the sequence. Compared to the pattern discovery method of [43], our method uses a more sophisticated scheme for whole-sequence scoring that has a formal probabilistic interpretation. We should note, however, that most of the patterns discovered by [43] were captured by our pHMMs (Figure 1), but in a more mathematically sound and exploitable manner. In addition, in [43] no overall measures of accuracy were reported in order to assess a fair comparison. Finally, our method is the only method reported until now, that is publicly available through a web-server. At the URL: , the user may submit a sequence in Fasta format, and receive the prediction. The method is rather fast, producing a self-explanatory output, and, thus it may be used by both molecular biologists requesting information for a single GPCR, and by bioinformaticians performing large-scale computational analyses.

At the final stages of preparation of this manuscript, another method developed independently by Sreekumar and coworkers, was published [52], which uses also pHMMs. However, the method of Sreekumar and coworkers, does not treat the multiple intracellular loops of a given GPCR independently, but instead it concatenates them into a single sequence. These concatenated sequences are then used to build pHMMs with the HMMER package. Although, the method performs very well as reported by the authors (they claim a 99% correct classification rate in a cross validation test), there are some severe disadvantages arising from the aforementioned strategy: With this method, to test a newly found protein, one has to perform predictions on the GPCR regarding its transmembrane topology, extract the intracellular loops and concatenate them into a single sequence. This adds another source of error, originating from the prediction errors of the transmembrane topology prediction algorithm. Having in mind, that to date, even the best topology prediction algorithms, predict correctly the full topology of a protein with an accuracy of no more than 75% [53,54], this will further reduce the performance of the method. We should also note that regarding GPCRs, the most accurate predictors fail to even predict seven transmembrane segments for more than 15% of the presented examples [54]. Furthermore, the method does not control appropriately the level of false positives, since it was not tested on non-GPCR sequences. On the contrary, the method proposed in this work, although it uses essentially the same principles in extracting the loop regions, it treats them independently using the Qfast algorithm, and thus, in the prediction phase, no a-priori knowledge of loops and transmembrane topology is needed. In addition to that, the rate of false positive predictions is controlled, providing us a confidence about the validity of the results. Last, and perhaps more important, our method is the only one until now that is fully automated and publicly available via a web-server.

## Conclusion

We applied here, a data-mining exploratory approach combined with the high discriminative power of profile Hidden Markov Models (pHMMs), to generate a system that predicts GPCR coupling specificity to the three main subfamilies of G-proteins (G_i/o_, G_q/11 _and G_s_), based solely on the information included in the protein sequence. We report superior correct classification rate compared to other previously published methods, and we have created a web-server, running the application, freely available for academic users (Commercial users should contact Professor S. J. Hamodrakas to obtain a licence). At present, this is the only web-based server for prediction of GPCRs coupling to G-proteins. Expanding this information to characterize the coupling properties for thousands of orphan GPCRs in large-scale proteome annotation studies, our understanding of receptor signaling pathways might improve and new targets for drug research may be uncovered. Future studies, utilizing larger representative training sets of GPCRs with known coupling specificity to G-proteins, and more advanced algorithmic techniques are needed in order to increase the accuracy of the prediction method, as also as to handle more efficiently the promiscuity in preferential coupling of GPCRs to G-proteins. This way, we may also be capable of predicting the coupling of GPCRs to G-proteins members of the G_12/13 _subfamily, a feature neither addressed in this study, nor in previously published methods.

## Methods

### Datasets

Our primary training dataset consists of 282 sequences of GPCRs of known coupling properties to G-proteins (120 G_i/o_, 94 G_q/11 _and 68 G_s_) according to the Trends in Pharmacological Sciences 2000 Receptor and Ion Channel nomenclature supplemement [37]. All sequences in the training dataset were of GPCRs with non-promiscuous coupling according to [36] and were retrieved from the Uni Prot 1.10 database [45], excluding fragments. Based on their coupling preference, they were grouped into Gi/o, Gq/11 or Gs coupled receptors. The Uniprot Accession numbers of the proteins in the training set can be found in our web-page . Moreover, an alternative non-redundant dataset comprised only of the 104 human counterparts of GPCRs in the original dataset was used to train the method. This was done in order to investigate the effect of redundancy posed by homologous sequences. A validation set was also generated, including 479 GPCR species homologues of the receptor subtypes with known coupling specificity according to [37] (256 G_i/o_, 102 G_q/11 _and 121 G_s_). Finally, the method was also validated on an independent set, composed of GPCRs, belonging to different subtypes with known coupling properties extracted from the gpDB database [18] that are not included in the training set [37].

As mentioned above, a sufficient amount of experimental data signifies the role of GPCR intracellular regions (the three intracellular loops and the carboxyl terminal region) and the membrane proximal intracellular extensions of transmembrane *α*-helices (approximately 1.5 turns) as the main regions of interaction between the G-proteins and the activated receptor complex [5]. Based on this experimentally derived information as well as membrane topology information derived from the UniProt annotation of each entry (in the "FT TRANSMEM" lines), we adapted our primary dataset, extracting sequence regions that corresponded to intracellular regions or transmembrane regions with intracellular proximity, spanning for approximately 7 residues within the cell membrane.

In order to investigate the performance of the method when applied to non-GPCR sequences, we used two alternative datasets. The first dataset includes a total of 1361 non-GPCR transmembrane receptors, whereas the second includes 1239 non-homologous globular proteins with structures known at atomic resolution [8].

### Data mining: Generation and evaluation of HMMs

A multiple alignment was generated for each group of intracellular sequence regions derived from GPCRs with same coupling preference using the ClustalX package [55]. Pairwise alignment scoring parameters were set as: BLOSUM 30 substitution matrix, Gap-opening penalty of 10.00 and gap extension penalty of 0.10. Multiple alignment parameters were set as BLOSUM 30 series substitution matrix, gap-opening penalty of 10.00 and gap extension penalty of 0.20. Multiple sequence alignments were then scanned for low entropy regions of high scoring alignment rows. Thus, the training dataset was further diminished to low-entropy sequence regions with a sequence identity criterion. The resulting multiple alignment rows were then used to generate a library of HMMs in an explorative way: for a given multiple alignment low entropy block starting from every offset and with any window of seven or more alignment rows a HMM was constructed. Thus, a low entropy block of n alignment rows generates



potential alignments, where w is the window length. This analysis yielded a total of 6149 HMMs that were tested on the fly with the *hmmsearch *program against the training set, in order to compare the discriminative power among alternative HMMs. As an estimator of the discriminative power of HMMs we calculated the Coverage of the results, i.e. the percentage of positives scoring an e-value lower than the lowest e-value of the negatives. This critical e-value corresponds to the noise cutoff of Pfam entries [51]. In order to compare Coverage values between HMMs derived from different coupling groups, we calculated the p-value of Coverage as a random variable (probability of a model derived from alignment rows of random sequences scoring a Coverage greater or equal to a specific observed Coverage), as follows:

In a set of n sequences containing *κ *positives, the probability of choosing *x *positives before the first negative, without reset, equals to:



where *f(x) *is the probability function of the negative hypergeometric distribution. The cumulative probability function, i.e. the probability of choosing *x *or less positives before the first negative without reset is:



Thus, the probability of choosing × or more positives before the first negative (i.e. the p-value of the test) equals to 1 - *F*(*x*).

**Figure 2:**
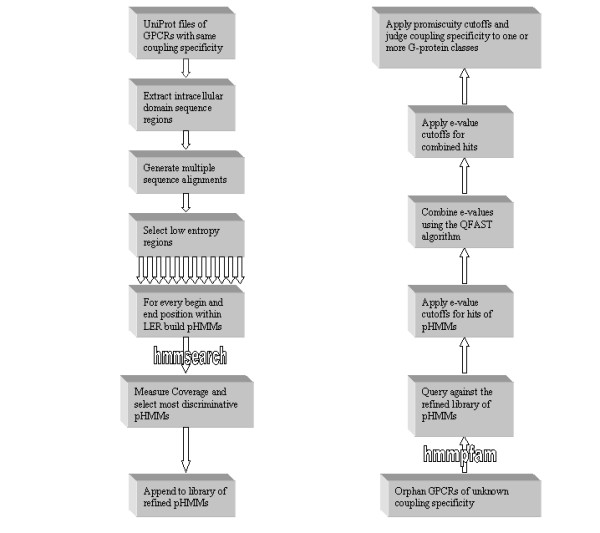
Flow chart of the method. GPCR entries in UniProt of known, non-promiscuous coupling specificity to G-proteins summarized in [37] were used to extract intracellular regions sequences, based on membrane topology information of the UniProt annotation. ClustalX was used to generate multiple sequence alignments of intracellular regions from which low-entropy blocks were selected based on ClustalX row scores. For every begin and end row, within low-entropy regions, sub-alignments were extracted and profile Hidden Markov models (pHMMs) were built. The discriminative power of each pHMM was assayed, after an hmmsearch run against the training set. The most discriminative HMMs for each intracellular region were selected for each one of the three main coupling groups and appended in the refined library. E-value thresholds were then set for each pHMM included in the refined library. The reverse course is followed during a query against the library of refined pHMMs.

Based mainly on Coverage measurements and their p-values, we discovered HMMs from several sub-regions within the loops that show up to 12-fold increase of Coverage, in comparison to models derived from the entire loop sequences. Our exploratory approach resulted in 5 to 7 refined pHMMs for each one of the three groups of GPCRs in the dataset, as summarized in Table 4. The overall flow chart of the method, is presented in Figure 2.

### Cutoff optimisation

**Figure 3:**
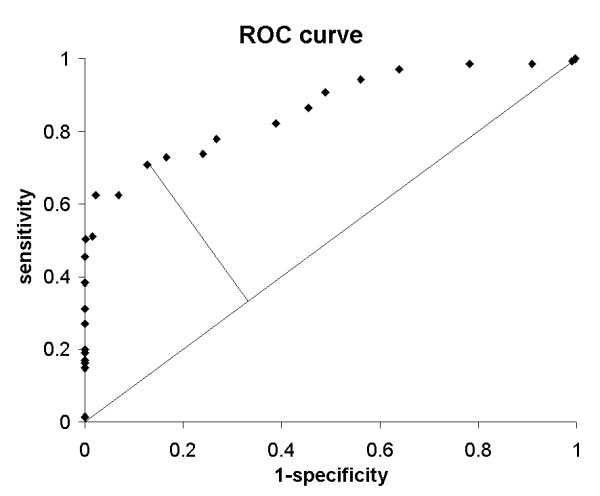
ROC curve analysis. ROC curve as an assay of discriminative power of a profile Hidden Markov models (pHMMs) that models a sub-region within the second intracellular loop of GPCRs that are known to couple with G-proteins of the G_s _subfamily. The space under the ROC curve is a measure of the discriminative power of the model, while the distance of each point from the diagonal of the chart y = x is a measure of combined specificity and sensitivity of the model. The e-value that corresponds to the maximal distance from the diagonal spot is set as the threshold that discriminates positive from negative predictions in an hmmpfam run, regarding the selected pHMM. Similar charts were applied to optimize e-value cutoffs of all HMMs in the refined final library.

Due to sequence similarity among intracellular regions of GPCRs of different coupling preferences, an *hmmpfam *query of the training set against the refined HMM library under a default e-value cutoff threshold, yielded artifact promiscuity in the predictions. HMMs derived from different intracellular regions show extensive variation in their discriminative power, as a consequence of different participation of different regions among the three groups of GPCRs in the overall coupling preference of the molecule. Our aim was not to exclude promiscuity from the predictive power of the method, so each HMM in the refined library was given a discrete cut off threshold resulting from ROC curve analysis after evaluation of the distribution of scores that corresponded to positive and negative targets of the training set. For each refined HMM of our library, we estimated and applied cutoff values that maximize the value:, where TP and FP are the percentages of positives and negatives respectively that score an e-value equal or less than a defined threshold at an hmmpfam query against the training set. This value is represented by the distance from the diagonal *f(x) *= *x *of a ROC curve, as presented in Figure 3. Once we had estimated the optimized cutoff thresholds for each HMM fingerprint, we combined the e-values of queries against HMMs that characterize the same GPCR coupling group using the QFAST algorithm [56]: , where *p *is the maximum product of *n *independent, uniform random variables and *F*(*p*) the combined p-value. In order to maximize the discriminative power of the combined predictions, a cutoff threshold was set for each one of the three e-value combinations, based mainly on the shape of the distribution of e-values scored by positives and negatives of the test set. A threshold was also set for the difference of combined e-values scored by the first and second match, expressed in logarithmic units, based on the combined e-values scored by HMMs characterizing different coupling groups in a *hmmpfam *search against a set of all 24 coupling-promiscuous GPCR sequences summarized in [37]. This estimation was based on the observation that the distributions of combined e-value differences, between the first two coupling predictions, from queries against promiscuous and non-promiscuous GPRCs are distinguishable when expressed in a logarithmic scale. Thus, alternative coupling groups are predicted as multiple combined e-value hits when querying the library against GPCR sequences of promiscuous coupling properties.

In order not to over-estimate the correct classification rate, a five-fold cross-validation procedure was adopted. Initially, the training set was randomly divided to five equally balanced sets. Afterwards, we trained a model, according to the above-mentioned procedure, using as training set the sequences in the four sub-sets, whereas the last sub-set was used for testing. This procedure was repeated five times, and the final results are the overall results obtained from the five sets.

## Authors' contributions

NS performed the analysis, and implemented the algorithms and the web-interface. PB formulated the problem, collected the training and testing sets and designed the training scheme. PP implemented the Qfast algorithm and participated in the optimization procedure. SH coordinated and supervised the project suggesting innovative features. NS, PB and SH drafted the manuscript. All authors have read and accepted the manuscript.

## Figures and Tables

**Table 1:** Results of the cross-validation and independent set tests. **A**. Correct classification rate results obtained from the three main G-protein coupling groups, in a five-fold cross-validation procedure. The training set was randomly divided to five equally balanced sets. Afterwards, we trained a model using the sequences in the four sets whereas the last set was used for testing. This procedure was repeated five times. **B**. The library of refined profile Hidden Markov models (pHMMs) derived from the primary dataset of 282 GPCRs (see text) was tested against a validation set comprised of all GPCR sequences of subtypes with known coupling preference summarized in [37], excluding the sequences used to train the models (479 GPCRs in total). This independent test yielded 91% correct classification rate. Numbers in the diagonal of the charts represent true positive predictions. The total number of predictions for each group (row) is not equal to the total number of observations, since several GPCRs were not classified in any group.

**A**	**predicted**				
*Five-fold cross-validation test*		**G_i/o_**	**G_q/11_**	**G_s_**	**total**

**observed**	**G_i/o_**	109(90.8%)	1	1	120
	**G_q/11_**	2	78(82.9%)	2	94
	**G_s_**	0	0	66(97.1%)	68
		111	79	69	253(89.7%)

**B**	**predicted**				

*Validation test (479 GPCRs)*		**G_i/o_**	**G_q/11_**	**G_s_**	**total**

**observed**	**G_i/o_**	233(91.4%)	16	4	256
	**G_q/11_**	9	90(88.2%)	2	102
	**G_s_**	6	2	113(93.4%)	121
		248	108	119	436(91.0%)

**Table 2:** Prediction results for 30 different GPCR subtypes with known coupling properties extracted from the gpDB database [18] that are not included in the training set [37]. The annotation of GPCR coupling properties in gpDB is based on data from the scientific literature. According to the gpDB classification scheme, G_gust _and G_t _G-proteins belong to the G_i/o _subfamily and G_olf _to the G_s _subfamily. The GPCR sequences were extracted from gpDB and parsed into our prediction server .

**GPCR Subtype**	**Uniprot AC**	**observed**	**predicted**
5-OXO-ETE receptor TG1019	Q8TDS5	G_i/o_	G_q/11_
Allatostatin receptor	Q9W4R0	G_i/o_	G_i/o _G_q/11_
Apelin receptor	P35414	G_i/o_	G_i/o_
Gonadotropin releasing hormone I receptor	Q92644	G_q/11_	G_i/o_
Gonadotropin releasing hormone II receptor	Q8TCX8	G_q/11_	G_q/11_
Gustatory receptor 43	Q9JHE2	G_gust_	G_i/o_
Gustatory receptor GUST27	P34987	G_i/o_	G_i/o_
Neuromedin U1 receptor	Q9JIB2	G_q/11_	G_q/11 _G_i/o_
Neuromedin U2 receptor	Q9NRA6	G_q/11_	G_q/11 _G_i/o_
Nicotinic Acid receptor	Q8TDS4	G_i/o_	G_i/o_
Olfactory receptor 10A7	Q96R19	G_olf_	G_s_
Blue opsin	Q13877	G_t_	G_i/o_
Orexin 1 receptor	O43613	G_q/11_	G_i/o _G_q/11_
Orexin 2 receptor	O43614	G_q/11_	G_i/o _G_q/11_
Platelet activating factor receptor	P25105	G_i/o _G_q/11_	G_i/o_
Prolactin-releasing peptide receptor	O75194	G_q/11_	G_i/o_
Trace amine receptor TAR	Q9P1P4	G_s_	G_s_
Trace amine receptor TAR-1	Q96RJ0	G_s_	G_q/11 _G_s _G_i/o_
Trace amine receptor TAR-2	Q923Y7	G_s_	G_s _G_i/o_
Trace amine receptor TAR-3	Q96RI9	G_s_	G_i/o _G_s_
Urotensin II receptor	Q9UKP6	G_q/11_	G_i/o_
Gastric inhibitory peptide receptor	Q9UPI1	G_s_	G_s_
Glucagon Receptor	P47871	G_s_	G_s_
Glucagon like peptide receptor 2	O95838	G_s_	G_s_
Ghrelin receptor	Q96RJ7	G_q/11_	G_q/11_
Parathyroid hormone receptor 1	P49190	G_s_	G_s_
Parathyroid hormone receptor 2	Q03431	G_s_	G_s_
Secretin receptor	Q13213	G_s_	G_s_
Calcium-sensing receptor	P41180	G_i/o _G_q/11_	G_i/o_
Taste receptor TASTE_TB334	Q62942	G_gust_	G_i/o_

**Table 3:** Promiscuous validation test. All 24 sequences of promiscuous coupled GPCR subtypes (as summarized in [37] and other sources [38]) were parsed in an hmmpfam query against our refined library of profile Hidden Markov models (pHMMs). The query sequences were not included in the training set. Promiscuous coupling was correctly predicted for 6 queries.

**GPCR Subtype**	**Uniprot AC**	**observed**	**predicted**
Alpha-2A adrenergic receptor	P08913	G_q/11 _G_i/o _G_s_	G_q/11 _G_i/o _G_s_
AT1 angiotensin receptor	P30556	G_q/11 _G_i/o_	G_q/11 _G_i/o_
Beta-3 adrenenergic receptor	P25962	G_i/o _G_s_	G_q/11 _G_io _G_s_
C3A anaphylatoxin chemotactic receptor	Q16581	G_q/11 _G_i/o_	G_i/o_
Calcitonin receptor	P30988	G_q/11 _G_s_	G_s_
Cholecystokinin type 1 receptor	P32238	G_q/11 _G_s_	G_q/11 _G_i/o_
Sphingosine 1-phosphate receptor 3 (EDG3)	Q99500	G_q/11 _G_i/o_	G_i/o_
Lysophosphatidic acid receptor 2 (EDG4)	Q9HBW0	G_q/11 _G_i/o_	G_i/o_
Endothelin B receptor	O60883	G_q/11 _G_i/o_	G_i/o_
Endothelin A receptor	P25101	G_q/11 _G_s_	G_q/11_
Follicle-stimulating hormone receptor	Q95179	G_i/o _G_s_	G_s_
Galanin receptor type 2	O43603	G_q/11 _G_i/o_	G_q/11 _G_i/o_
Leukotriene B4 receptor	Q15722	G_q/11 _G_i/o_	G_q/11 _G_i/o_
Lutropin-choriogonadotropic hormone receptor	P22888	G_q/11 _G_i/o _G_s_	G_s_
Neuromedin K receptor (NK-3)	P29371	G_q/11 _G_s_	G_q/11 _G_i/o_
Neuropeptide Y receptor type 1	O02813	G_q/11 _G_i/o_	G_i/o_
Oxytocin receptor	P30559	G_q/11 _G_i/o_	G_q/11 _G_i/o_
P2Y purinoceptor 11	Q96G91	G_q/11 _G_s_	G_i/o_
Platelet-activating factor receptor	Q62035	G_q/11 _G_i/o_	G_q/11 _G_i/o_
Prostaglandin E2 receptor EP3	P43115	G_q/11 _G_i/o _G_s_	G_i/o_
Substance-K receptor (NK-2)	P21452	G_q/11 _G_s_	G_q/11 _G_i/o_
Substance-P receptor (NK-1)	P25103	G_q/11 _G_s_	G_q/11 _G_i/o_
Proteinase activated receptor 1 (thrombin receptor)	P25116	G_q/11 _G_i/o_	G_i/o_
Thyrotropin receptor	P47750	G_q/11 _G_i/o _G_s_	G_i/o _G_s_

**Table 4:** Fingerprint discovery results. Coverage values of profile Hidden Markov models (pHMMs) given after each alignment of entire loop sequence regions in comparison to maximized Coverage values of highly discriminative pHMMs derived from selected sub-alignments within low-entropy regions. pHMMs derived from sub-alignments showed up to 12-fold increase in their discriminative power, as measured by Coverage, in comparison to pHMMs that characterize the entire loop sequence alignments. Alternative alignment regions with the same discriminative power are separated by commas, double dots separate beginning and ending of sub-alignments used to generate HMMs. In the case of discovery of non-overlapping sequence fragments with high discriminative power (e.g. G_s _loop1), separate pHMMs were generated and appended in the refined library of that group.

Coupling preference	G_i/o_	G_q/11_	G_s_
Intracellular regions	loop1	loop1	loop1
Whole sequence region	1..39/2.65	1..28/16.05	1..59/58.01
Non-overlapping most discriminative regions	3..24/19.86	13..25/30.53	7..19/63.35,43..51/48.09
			
	loop2	loop2	loop2
	1..50/19.20	1..42/0.70	1..39/12.21
	10..18/32.78	14..34/19.85	18..25/54.19
			
	loop3a	loop3a	loop3a
	1..29/19.54	1..17/16.79	1..15/27.48
	2..18/27.15	5..17/19.08	6..15/42.74
			
	loop3b	loop3b	loop3b
	1..26/6.63	1..21/3.82	1..15/20.61
	14..21/23.51	8..19,9..20/12.21	1..15/20.61
			
	loop3c	loop3c	loop3c
	1..29/8.28	1..17/3.81	1..22/0.76
	8..21/36.09	2..12/48.85	8..21,13..21/10.68
			
	C-terminal	C-terminal	C-terminal
	1..29/8.28	1..24/12.97	1..29/29.77
	8..21/36.09	5..19/35.87	8..16/57.25
